# Application of MRI images based on Spatial Fuzzy Clustering Algorithm guided by Neuroendoscopy in the treatment of Tumors in the Saddle Region

**DOI:** 10.12669/pjms.37.6-WIT.4850

**Published:** 2021

**Authors:** Peng Zhang, Lingdang Zhang, Rui Zhao

**Affiliations:** 1 Peng Zhang, Attending Physician. Department of Neurosurgery, Chongqing Three Gorges Central Hospital, 165 Xincheng Road, Wanzhou District, Chongqing, 404100, China; 2 Lingdang Zhang, Attending Physician. Department of Neurosurgery, Chongqing Three Gorges Central Hospital, 165 Xincheng Road, Wanzhou District, Chongqing, 404100, China; 3 Rui Zhao, Associate Chief Physician. Department of Neurosurgery, Chongqing Three Gorges Central Hospital, 165 Xincheng Road, Wanzhou District, Chongqing, 404100, China

**Keywords:** Spatial fuzzy clustering algorithm, Neuroendoscopy, Tumors, Saddle region, Complications

## Abstract

**Objective:**

The paper applies spatial fuzzy clustering algorithm to explore the role and value of neuroendoscopic assisted technology in the operation of tumors in the saddle region, and analyze the MRI image characteristics of tumors in the saddle region.

**Methods:**

The clinical data of 63 patients from our hospital who underwent neuroendoscopic assisted microscopy to remove tumors in the saddle area from 2017 to 2019 (neuroendoscopy-assisted group) were collected. Seventy six patients who occupied the saddle area by microscopic resection only in the same period (Simple microscope group) clinical data. By comparing the patient’s tumor resection rate, postoperative complication rate and postoperative recurrence rate, the surgical effect was evaluated.

**Results:**

The total resection rates of the tumors in the neuroendoscopy-assisted group and the microscope-only group were 95.24% (60/63) and 80.26% (61/76). The incidence of postoperative vasospasm was 3.17% (2/63) and 13.16% (10/76), the incidence of nerve injury was 0 (0/63) and 6.58% (5/76), the difference was statistically significant (P <0.05). There was no significant difference in the incidence of postoperative infection, cerebrospinal fluid leakage and postoperative recurrence rate between the two groups (P> 0.05).

**Conclusion:**

Neuroendoscopy-assisted microscopy-based removal of the saddle area occupying space based on spatial fuzzy clustering algorithm can increase the total tumor resection rate and reduce the incidence of complications.

## INTRODUCTION

The reasonable use of endoscope-assisted microscope can reduce trauma and effectively improve the rate of total tumor resection. At present, only some researchers have made some clinical attempts. The comparison of the efficacy of neuroendoscopic assisted surgery and simple microsurgery is relatively small, especially total tumor resection., The incidence of postoperative complications and postoperative recurrence rates are lacking in comprehensive research. We collected the clinical data of 63 patients who underwent neuroendoscopic assisted microscopy to remove tumors in the saddle area in our hospital from 2015 to 2017, and the clinical data of 76 patients who occupied the saddle area by microscope removal from 2013 to 2015 A systematic and comprehensive controlled study of neuroendoscopy-assisted and conventional microsurgery for the treatment of saddle space occupying is reported below.^1^,^2^

## METHODS

All 139 patients in the group were from patients with tumors in the saddle area that were removed by the same surgeon. They all have indications for surgery, no serious underlying diseases of all systems in the whole body, and no contraindications to surgery. Among them, 63 patients who underwent neuroendoscopy-assisted microscopy to remove tumors in the saddle region (neuroendoscopy-assisted group), 33 males and 30 females, aged (52.7 ± 16.7) years old, tumor size 1.5-6.3 cm; 32 meningiomas. There were 18 cases of craniopharyngioma, 10 cases of invasive pituitary tumor, 1 case of germ cell tumor, and 2 cases of epidermoid cyst; the course of disease was 10d to 6 years; and the length of hospitalization was 10 to 28d. Only 76 cases of the saddle area were removed by microscope (simple microscope group), 38 males and 38 females, aged (53.6 ± 15.8) years old, tumor size 1.8-6.3 cm; 42 meningiomas and 20 craniopharyngiomas, 10 cases of invasive pituitary tumors, 2 cases of germ cell tumors, and 2 cases of epidermoid cysts; the course of disease was 13 days to 4 years; the length of hospitalization was 8-40 days. There was no statistically significant difference in gender composition, age, tumor type, course of disease, and length of hospital stay between the two groups (P> 0.05), which was comparable. This study was reviewed by the ethics committee of our hospital.^3^,^4^

### (2) Surgical methods

All patients choose appropriate surgical approaches such as subfrontal lateral, frontal longitudinal fissure, wing point, corpus callosum-vault, etc. according to the nature, size, growth direction, and degree of invasion of the disease before surgery. Craniotomy was performed under general anesthesia of tracheal intubation. During surgery, the microscope was first used to remove the tumor, until the tumor could not be seen or could not be removed. The neuroendoscopic auxiliary group then placed the neuroendoscopy to observe and inspect the dead angle of the visual field. If there was any residual tumoradjust the microscope angle until the tumor is completely removed and close the skull routinely.^5^,^6^

### Observation indicators

The observation indicators include intraoperative observation indicators, observation indicators within 1 week after operation, the incidence of postoperative complications, and observation indicators of long-term efficacy after operation.

### MRI examination

All patients underwent plain scan or enhanced scan with MRI instrument (TOSHIBAVISART / EX1.5T); among them, 11 cases were plain scan, 41 cases were enhanced scan; conventional coronal, sagittal and axial scan T1WI, TR / TE = 500 / 20ms; axial position T2WI, TR / TE = 2000 / 80ms; sagittal plane, coronal plane and cross-section scan layer thickness are 5mm; patients with enhanced scan are injected with contrast agent magnetic imaging meglumine (Gd-DTPA) 0.1 mmol / kg, half-injection was performed for coronal and sagittal scanning, and then full-enhanced scanning. Imaging analysis All patients’ MRI examination imaging data are determined by more than four imaging practitioners and neurosurgery practitioners combined with clinical manifestations to determine the tumor type.

### Statistical methods

The paper uses SPSS19.0 software to process the data, and the measurement data in accordance with the normal distribution is expressed as x ± s, the comparison is by t test, the comparison between the count data is by χ^2^ test, and P <0.05 is considered statistically significant.

## RESULTS

Intraoperative monitoring records and statistics of the neuroendoscopic auxiliary group is shown in. Table-I. Intraoperative neuroendoscopic tumor resection in 60 cases, subtotal resection in 2 cases, most cases in 1 case, endoscopic assisted resection in 13 cases, endoscopic assisted total resection in 10 cases, total resection rate was 95.24% (60/63).

**Table-I:** Intraoperative monitoring records and statistics of the neuroendoscopic auxiliary group (cases).

*Tumor*	*0 Minute*	*1 Minute*	*2 Minute*	*3 Minute*	*4 Minute*	*5 Minute*
Meningioma	26	3	1	1	1	0
Craniopharyngioma	15	1	1	1	0	0
Aggressive pituitary tumor		9	1	0	0	0
Germ cell tumor		0	1	0	0	0
Epidermoid cyst	0	1	1	0	0	0

Two groups of patients underwent brain CT and MRI scans and enhanced scans to check tumor resection. There was no significant difference in the resection of craniopharyngioma, invasive pituitary tumor, germ cell tumor, and epidermoid cyst between the two groups (P> 0.05). However, the rate of total tumor resection [95.24% (60/63) vs. 80.26% (61/76)] and meningiomas [93.75% (30/32) vs. 78.57% (33/42)] The difference is statistically significant (P <0.05).

***(3)*** Comparison of the incidence of postoperative complications between the two groups. See Table-II. There was no statistically significant difference in the incidence of postoperative electrolyte disturbance, cerebrospinal fluid leakage, and infection between the two groups (P> 0.05), and there was a statistically significant difference in the incidence of postoperative vasospasm and nerve damage (P<0.05), assisted by neuroendoscopy Postoperative vasospasm occurred in the group of 3.17% (2/63) and nerve injury was 0 (0/63); in the simple microscope group, vascular spasm occurred in 13.16% (10/76) and nerve damage was 6.58% (5/76).

**Table-II:** Comparison of the incidence of postoperative complications between two groups of patients with tumors in the saddle area (cases).

*Group*	*Electrolyte*	*Vasospasm*	*Nerve damage*	*Cerebrospinal fluid leakage*
Neuroendoscopic auxiliary group	9	2	0	0
Simple microscope group	17	10	5	1
χ^2^ value	1.48	4.35	4.3	0.83
P value	>0.05	<0.05	<0.05	>0.05

Comparison of recurrence rate at one year after operation between the two groups. One year later, 2 patients and three patients in the neuroendoscopic auxiliary group and the simple microscope group were lost to follow-up respectively. The follow-up patients’ tumor recurrence is shown in Table-III.

**Table-III:** Comparison of tumor recurrence at 1 year after operation between two groups of patients with tumors in the saddle area (cases).

*Group*	*Relapse*	*No recurrence*	*χ^2^ value*	*P value*
Neuroendoscopic auxiliary group	1	60	1.36	>0.05
Simple microscope group	4	69		

## DISCUSSION

In terms of the typical imaging features of tumors in the sellar region, tumors in the sellar region are mainly located in or above the sellar, and multiaxial scanning is easy to locate and characterize.^7^-^9^ For example, the presence and absence of an intrasellar pituitary gland can help distinguish suprasellar and intrasellar tumors, i. e. pituitary tumors. 10,11 However, suprasellar tumors often invade into the sellar and compress the pituitary to move backward, and the imaging appearance is crescent. Enhanced MRI usually shows the characteristic signs of sellar region tumors, such as “meningioma tail sign”, which is the gold standard for the diagnosis of meningioma.^12^-^14^ Craniopharyngioma MRI enhanced scan can show the enhancement of the cyst wall, which has obvious calcification and is easy to determine. 15-16 Large pituitary adenoma usually presents “girt waist sign”,^17^,^18^ which has also been reported as “snowman sign”.^19^

Some typical features, such as internal carotid artery embedding sign and paranasal sinus tamponade sign, are also of great value in the qualitative diagnosis of sellar region tumors. Calcification is seen in most tumors in sellar region, so calcification is an important basis for qualitative diagnosis of sellar region tumors. MRI shows less calcification than CT, and it is difficult to detect fine calcification, while its three-dimensional imaging can accurately show the location and adjacent situation of the tumor. Contrast-enhanced MRI scan can further distinguish most tumors from adjacent blood vessels and tissues. The cyst wall of craniopharyngioma can be enhanced in a ring, which is helpful to the differential diagnosis of craniopharyngioma. Enhanced MRI scan of meningioma showed “meningeal tail sign”. MRI signals of pituitary adenoma were easily disturbed by calcification and bone signals, and enhanced scan showed “internal carotid artery embossing sign”. The enhancement time of pituitary microadenoma is earlier than that of normal pituitary, which is helpful for qualitative diagnosis.

Cystic tumors showed no significant enhancement, while solid tumors showed significant enhancement on MRI. T1WI images showed uneven high and low signal or uniform high signal. Therefore, it is difficult for MRI and contrast-enhanced scanning to identify suprasellar solid tumors. In this study, pituitary tumor was misdiagnosed as solid craniopharyngioma, with a high misdiagnosis rate. Therefore, the diagnosis rate of solid deformed tumor should be improved by combining histopathology and related endocrine detection. MRI can provide multi-directional images with high contrast, clear image, and little artifact interference. It can also show the relationship between sellar tumors and adjacent tissues and blood vessels. The results showed that MRI could not be accurately diagnosed, which might be related to too small sample size or inexperience of the viewers. Nevertheless, MRI can be used as the preferred imaging diagnostic method. In summary, there are various imaging manifestations of sellar tumors, and MRI can well display the relationship between sellar tumors and adjacent tissues and blood vessels. Multidirectional MRI scanning was used to fully understand the imaging characteristics of sellar region tumors. Only by combining relevant pathology, serological examination and endocrine examination, can the accuracy of diagnosis of sellar space occupying be improved and misdiagnosis be reduced.^20^

### Limitations of the study

Due to the limitations of MRI itself, the appropriateness of the front view positioning film during scanning is related to whether the front view color coded image is clearly presented in the same plane. The correct selection of points of interest is directly related to the accuracy and clarity of the white matter fiber bundle imaging in the post-processing of MRI images. Therefore, the technology has a certain subjective dependence on the operator. Due to the small number of cases included in this study, some conclusions were drawn, especially in the process of statistical analysis, which was limited and influenced to some extent. Moreover, since the patients included in this study were only followed up for a short time, the lack of long-term follow-up results also affected the corresponding results.

## CONCLUSIONS

In short, proficiency in neuroendoscopy and intraoperative auxiliary microscope removal of tumors in the saddle area can not only improve the total tumor resection rate, but also reduce postoperative complications, which is more conducive to patient prognosis.

### Author`s Contribution

**PZ** conceived the study, literature review, data analysis, drafting the manuscript.

**LZ** helped in design, data collection, & critical revision.

**RZ** takes the responsibility and is accountable for all aspects of the work in ensuring that questions related to the accuracy or integrity of any part of the work are appropriately investigated and resolved.
